# Calcific tendinitis of an extensor tendon misdiagnosed as stenosing tenosynovitis: A case report

**DOI:** 10.1016/j.jpra.2026.04.021

**Published:** 2026-05-02

**Authors:** Chengtian Xiao, Degen Tian, Xiaohua Liang, Simin Li, Fan Chen, Haoxiong Chen, Qiliang Xu

**Affiliations:** a Department of Orthopaedic Surgery, Shenzhen Hospital (Futian) of Guangzhou University of Chinese Medicine, 6001 Beihuan Avenue, Futian District, Shenzhen, Guangdong, China; b Department of Gynecology, Guangxi University of Chinese Medicine, 179 Mingxiu East Road, Xixiangtang District, Nanning, Guangxi, China

**Keywords:** Calcific tendonitis, Extensor tendon, Shockwave therapy, Extracorporeal shockwave therapy

## Abstract

**Objective:**

Calcific tendonitis refers to the pathological deposition of calcium salts in or around tendons, leading to local inflammation and pain. It is commonly seen in the shoulder region, and occurrences in the wrist or hand are uncommon. This case report aims to describe a patient with calcific tendonitis of the wrist extensor tendons and to highlight the successful use of extracorporeal shockwave therapy (ESWT) as a treatment.

**Case presentation:**

Calcific Tendonitis of Extensor Tendons was diagnosed both via X-ray and Computed Tomography (CT) and was successfully treated with shockwave.

**Results:**

After the shockwave treatment, the patient experienced significant improvement in symptoms. Key outcomes included: Immediate Relief: The patient reported a marked reduction in wrist pain right after the ESWT session, with improved ability to move the wrist without discomfort. Continued Improvement: Over the subsequent weeks, her pain continued to diminish and wrist function steadily returned to normal. There were no episodes of recurring pain or swelling during the recovery period. Follow-Up Outcomes: At the 2-month follow-up, the patient was completely pain-free and had full range of motion in the affected wrist. A follow-up X-ray showed that the calcific deposit was no longer evident. Importantly, there were no signs of any complications such as local tissue damage or fractures on the imaging evaluation, confirming the effectiveness of the treatment.

**Conclusion:**

This case illustrates that extracorporeal shockwave therapy can be a highly effective treatment for calcific tendonitis before resorting to invasive measures, even at an unusual site like the wrist extensors.

## Introduction

Calcific tendinitis is an inflammatory condition caused by calcium salt deposition. It commonly affects the rotator cuff but may rarely involve the wrist or hand. While calcific tendinitis in flexor tendons has been documented, extensor tendon involvement is scarce. In these uncommon locations, it may present with acute pain, swelling, tenderness, and restricted motion, and can be mistaken for gout, infection, or tenosynovitis.

Traditional treatments like NSAIDs, ice packs, and physical therapy are often ineffective, particularly for persistent calcification. Recently, shock wave therapy (ESWT), a noninvasive treatment, has shown promise, especially for calcific tendinitis and tendon lesions.^1^ Although ESWT is well-established for larger tendons (e.g., the shoulder), its use for wrist tendon calcifications remains unexplored. This represents a knowledge gap, as ESWT could provide a noninvasive method to break up calcium deposits and promote tendon healing, potentially avoiding injections or surgery.

We present a case of a 55-year-old female with calcific tendinitis of the extensor tendons of the fingers (a rare condition) and examine the therapeutic effects of shock wave therapy on this condition.

## Case description

**Figure 1:**
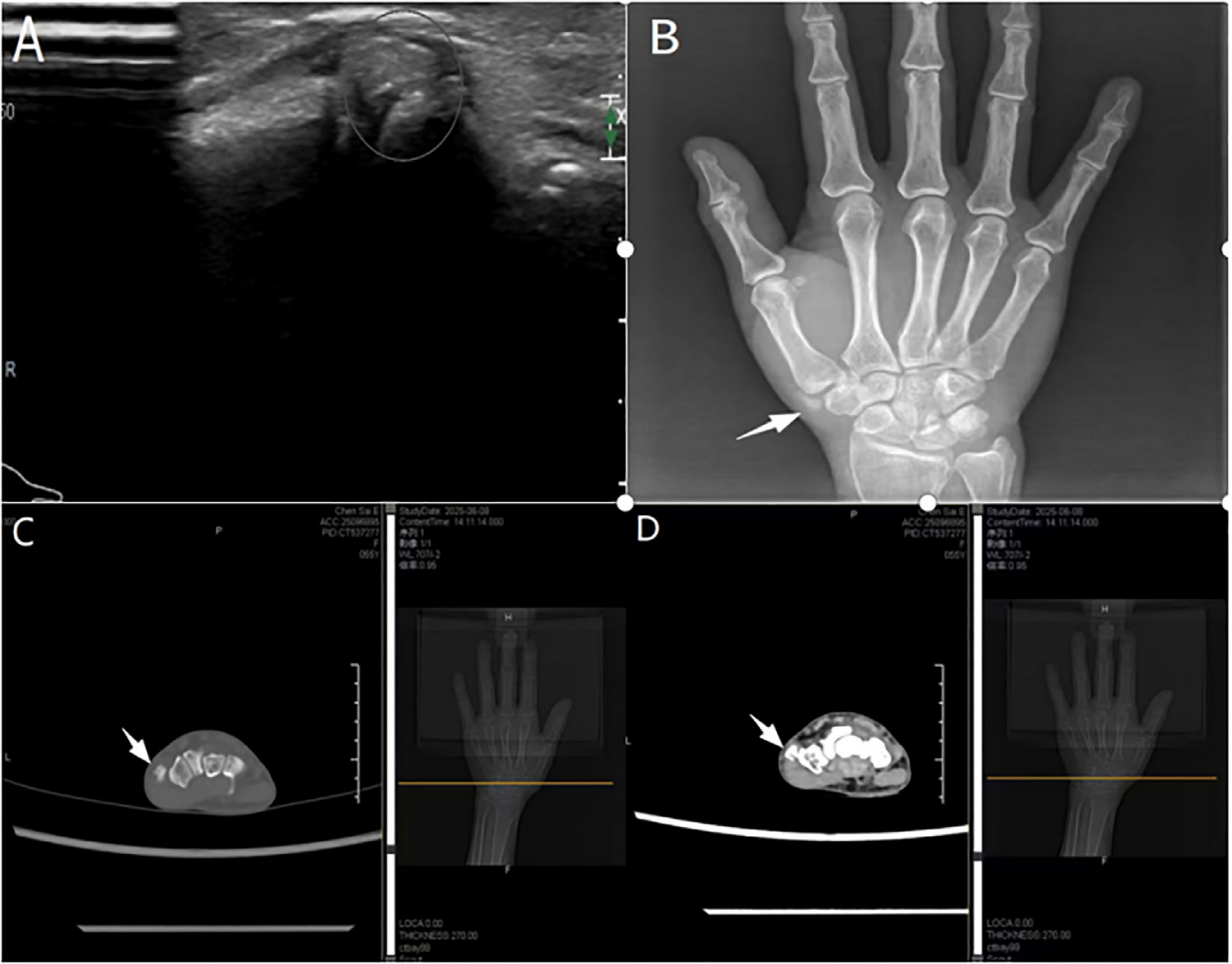
Before treatment radiographic findings. (A) Ultrasound image showing a hyperechoic mass (at the circle). (B) X-ray image showing well-defined calcification deposits in close proximity to the right extensor pollicis tendon (white arrow). (C) CT cross-sectional image of a window of bone displaying calcifications (white arrow). (D) CT cross-sectional image of a window of tissue displaying calcifications (white arrow).

A 55-year-old woman presented with a 4-day history of pain, redness, swelling, and warmth over the right radial styloid, accompanied by limited thumb motion. She had no history of trauma, sepsis, gout, or previous joint pain, and no family history of similar conditions. Physical examination revealed localized erythema and swelling over the radial aspect of the wrist, marked tenderness at the radial styloid, and a positive Finkelstein’s test. Because the radial-sided pain, swelling, and positive Finkelstein’s test were clinically compatible with de Quervain-like stenosing tenosynovitis, an initial diagnosis of stenosing tenosynovitis was made. However, her symptoms persisted despite treatment with topical plaster, prompting further evaluation. Ultrasonography demonstrated hyperechoic masses between the scaphoid and the first metacarpal joint with multiple strong echogenic foci (Figure 1A). Plain radiography revealed calcific deposits at the lateral margin of the first carpometacarpal joint (Figure 1B), and CT further confirmed calcific tendinitis (Figure 1C, D). Laboratory findings were within normal limits, and no evidence of systemic rheumatic disease was identified.

**Figure 2:**
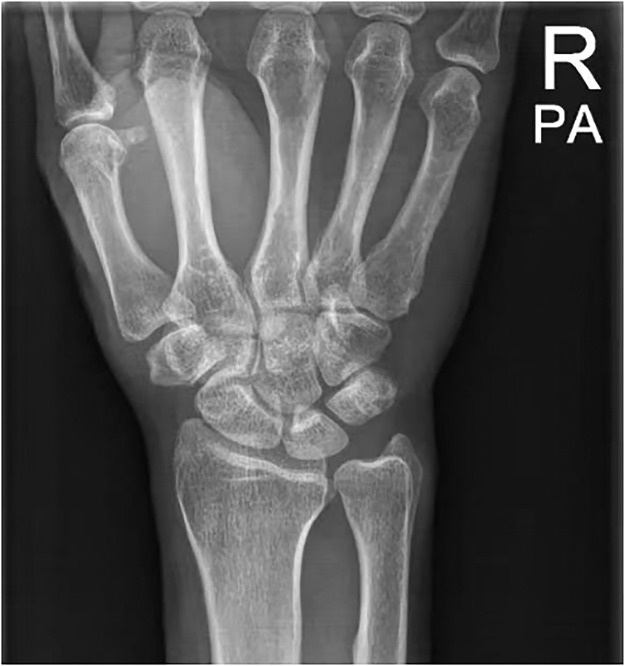
After treatment radiographic findings. X-ray image demonstrating complete debridement of calcific deposits.

The patient underwent ESWT on June 5, 2025, with 2000 impulses per session at 5–10 Hz and 2.0–2.5 bar, using low-to-medium manual contact pressure, for a total of 5 sessions at 7-day intervals. After the first session, pain decreased markedly and active thumb motion improved clinically. By the end of the fifth session, the calcific deposit was completely absorbed, and symptoms resolved. Two months post-treatment, the patient reported no pain, improved function, and regained normal wrist activity. Follow-up X-ray showed complete resolution of calcific deposits, and the wrist joint function had returned to normal (Figure 2).

## Discussion

Calcific tendinitis has an incidence rate of 2.5−7.5% in adults, with a slight female predominance (approximately 70%).^2^ The rotator cuff tendons, especially the supraspinatus tendon, are the most commonly affected sites, accounting for about 80% of cases.^3^ Although often seen in the shoulder, calcific tendinitis has been reported in other joints, such as the hip, elbow, wrist, and knee.^4^ The extensor tendons of the fingers, responsible for finger extension, are located on the dorsal side of the hand. To date, no cases of calcific tendinitis in the extensor tendons of the fingers have been reported, highlighting its rarity. While calcific tendinitis has been documented in flexor tendons (e.g., trigger finger), extensor tendon involvement remains extremely rare.

Calcific periarthritis or tendinopathy is a complex condition with an unclear cause. Modern understanding frames calcific tendinopathy as an active process, where repeated minor trauma or ischemia in tendon areas with poor blood supply triggers fibrocartilaginous metaplasia, allowing calcium deposition.^5^

Calcific tendinopathy can cause sudden, severe wrist pain and limited movement, often mimicking conditions like gout, pseudogout, septic arthritis, or severe tenosynovitis. It is frequently misdiagnosed, with about one-third of patients initially thought to have an infection or arthritis.^6^ Another common misdiagnosis is stenosing tenosynovitis, but calcific tendinitis tends to have a more acute and severe onset. In the present case, the positive Finkelstein’s test did not contradict the final diagnosis; rather, it contributed to the initial misdiagnosis. Because the calcific lesion was located adjacent to the radial styloid and involved the extensor tendon region related to thumb motion, provocative maneuvers reproduced a pain pattern similar to that seen in de Quervain-like stenosing tenosynovitis. This overlap explains why the initial clinical impression was misleading and highlights the importance of imaging in patients with acute radial-sided wrist pain and atypical evolution.

There is no universally recognized gold standard for treating calcific bursitis or tendinitis. As a self-limiting condition, it is typically managed with conservative treatments, including rest, activity modification, NSAIDs, analgesics, ice, and physiotherapy. However, persistent calcific deposits may require more targeted intervention.

Ultrasound-guided percutaneous injection (UGPI) for rotator cuff calcific tendinitis has shown better clinical outcomes than corticosteroid injections or shockwave therapy.^7^ However, UGPI is not suitable for deposits smaller than 5 mm or those with firm consistency. While surgical removal was once an option, it is invasive and reserved for severe cases. Arthroscopic procedures also require hospitalization and long rehabilitation. Recently, minimally invasive treatments like ultrasound-guided needle aspiration and shockwave therapy have been developed as alternatives to surgery for persistent calcifications and inflammation.

Given the lack of a standardized extracorporeal shock wave therapy (ESWT) protocol for calcific tendinitis at the wrist/extensor tendon, we used a low-to-medium intensity regimen adapted from published ESWT parameters^8^ for upper-extremity tendinopathy and adjusted it according to lesion location and patient tolerance. ESWT has been shown to effectively relieve pain and clear calcifications in tendinopathies beyond the shoulder, such as in plantar fasciitis and tennis elbow.^9^ Studies confirm that shockwave therapy can achieve similar outcomes to invasive treatments like needle lavage and arthroscopic surgery, but with less risk and invasiveness.^10^ Our case highlights ESWT as an effective, noninvasive treatment for wrist calcific tendinitis, eliminating the need for steroids or surgery. The favorable clinical and radiographic response observed in this patient suggests that ESWT may be a useful noninvasive option in selected cases of anatomically unusual calcific tendinitis.

This report has several limitations. First, validated patient-reported outcome measures, such as the PRWE or DASH questionnaire, were not prospectively collected, which limited the quantitative assessment of functional recovery. Second, no validated pain scoring system, such as the Visual Analogue Scale (VAS) or Numeric Rating Scale (NRS), was prospectively used to quantify pain severity at baseline, during treatment, or follow-up. Therefore, pain improvement was documented descriptively rather than quantitatively. Future reports should incorporate serial standardized pain assessments. Third, the diagnosis was made on clinical and imaging grounds without histopathological or aspiration-based confirmation of calcium salt deposition. Although tissue confirmation is not routinely required in typical cases, its absence limits diagnostic certainty in this anatomically unusual presentation.

## Conclusion

Calcific tendinitis involving the extensor tendon region at the radial aspect of the wrist may clinically mimic stenosing tenosynovitis, particularly when radial-sided pain and a positive Finkelstein’s test are present. In such cases, multimodal imaging is essential for accurate diagnosis. This case also suggests that extracorporeal shock wave therapy may be a useful noninvasive treatment option for anatomically unusual calcific tendinitis, although standardized pain and functional outcome measures should be incorporated in future reports.

## Ethics statement

Ethical approval for this study was waived by Shenzhen Hospital (Futian) of Guangzhou University of Chinese Medicine.

## Funding

National Natural Science Foundation of China (82205301); Shenzhen Natural Science Foundation (JCYJ20240813160702004) and University-Hospital Joint Fund Project of Guangzhou University of Chinese Medicine (GZYFT2024G09) provided funding for this study.

## Availability of data and materials

Patient data can be provided upon request.

## Author contributions

CX: Data curation, Writing-original draft. DT: Writing-review & editing. XL: Formal analysis, Writing-review & editing. SL: Investigation, Writing-review & editing. FC: Writing-review & editing.

## Consent for publication

Written informed consent was obtained from the patient for publication of this case report and any accompanying images.

## Declaration of competing interest

All the authors declare that they have no competing interests.
